# Copper-61 is an advantageous alternative to gallium-68 for PET imaging of somatostatin receptor-expressing tumors: a head-to-head comparative preclinical study

**DOI:** 10.3389/fnume.2024.1481343

**Published:** 2024-10-11

**Authors:** Tais Basaco Bernabeu, Rosalba Mansi, Luigi Del Pozzo, Raghuvir Haridas Gaonkar, Lisa McDougall, Anass Johayem, Milen Blagoev, Francesco De Rose, Leila Jaafar-Thiel, Melpomeni Fani

**Affiliations:** ^1^ Division of Radiopharmaceutical Chemistry, University Hospital Basel, University of Basel, Basel, Switzerland; ^2^ Department of Nuclear Medicine, University Hospital Zurich, University of Zurich, Zurich, Switzerland; ^3^ Nuclidium AG, Basel, Switzerland

**Keywords:** copper-61, somatostatin receptors, neuroendocrine tumors, PET, theranostics

## Abstract

**Background:**

Gallium-68 positron emission tomography (^68^Ga-PET) with the two registered somatostatin analogs, [^68^Ga]Ga-DOTA-Tyr^3^-octreotide ([^68^Ga]Ga-DOTA-TOC) and [^68^Ga]Ga-DOTA-Tyr^3^-octreotate ([^68^Ga]Ga-DOTA-TATE), where DOTA = 1,4,7,10-tetraazacyclododecane-1,4,7,10-tetraacetic acid, is routinely used for imaging of somatostatin receptor (SST)-expressing tumors. We investigated copper-61 (^61^Cu) as an alternative radiometal for PET imaging of SST-expressing tumors. Compared to gallium-68, copper-61 (t_1/2_ = 3.33 h, E*_β_*^+^_max_ = 1.22 MeV) can be produced on a large scale, enables late time point imaging, and has the therapeutic twin copper-67. Herein, DOTA-TOC and 1,4,7-triazacyclononane,1-glutaric acid-4,7-acetic acid (NODAGA)-TOC were labeled with copper-61 and compared with the clinically used [^68^Ga]Ga-DOTA-TOC.

**Methods:**

[^61^Cu]CuCl_2_ was produced from an irradiated natural nickel target. DOTA-TOC and NODAGA-TOC were labeled with [^61^Cu]CuCl_2_ in ammonium acetate buffer so to achieve a reaction pH of 5–6 and a temperature of 95°C for DOTA-TOC or room temperature for NODAGA-TOC. The radioligands were evaluated head-to-head *in vitro* using human embryonic kidney (HEK)-SST_2_ cells (affinity, binding sites, cellular uptake, and efflux) and *in vivo* using HEK-SST_2_ xenografts [PET/computed tomography (CT) imaging, biodistribution, and pharmacokinetics] and compared with [^68^Ga]Ga-DOTA-TOC, which was prepared using a standard procedure. Dosimetry estimates were made for [^61^Cu]Cu-NODAGA-TOC.

**Results:**

[^61^Cu]Cu-DOTA-TOC and [^61^Cu]Cu-NODAGA-TOC were prepared at an apparent molar activity of 25 MBq/nmol with radiochemical purities of ≥96% and ≥98%, respectively. *In vitro*, both presented a sub-nanomolar affinity for SST_2_ (IC_50_ = 0.23 and 0.34 nM, respectively). They were almost entirely internalized upon binding to SST_2_-expressing cells and had similar efflux rates at 37°C. *In vivo*, [^61^Cu]Cu-DOTA-TOC and [^61^Cu]Cu-NODAGA-TOC showed the same accumulation in SST_2_-expressing tumors. However, PET/CT images and biodistribution analyses clearly showed an unfavorable biodistribution for [^61^Cu]Cu-DOTA-TOC, characterized by accumulation in the liver and the abdomen. [^61^Cu]Cu-NODAGA-TOC displayed favorable biodistribution, comparable with [^68^Ga]Ga-DOTA-TOC at 1 h post-injection (p.i.). Notwithstanding, [^61^Cu]Cu-NODAGA-TOC showed advantages at 4 h p.i., due to the tumor retention and improved tumor-to-non-tumor ratios. The effective dose (2.41 × 10^−3^ mSv/MBq) of [^61^Cu]Cu-NODAGA-TOC, but also the dose to the other organs and the kidneys (9.65 × 10^−2^ mGy/MBq), suggested a favorable safety profile.

**Conclusion:**

Somatostatin receptor ^61^Cu-PET imaging not only matches the performance of ^68^Ga-PET at 1 h p.i. but has advantages in late-time imaging at 4 h p.i., as it provides improved tumor-to-non-tumor ratios. [^61^Cu]Cu-NODAGA-TOC is superior to [^61^Cu]Cu-DOTA-TOC *in vivo*. The use of the chelator NODAGA allows quantitative labeling with copper-61 at room temperature and enables the straightforward use of a kit formulation for simple manufacturing in medical centers.

## 1 Introduction

Positron emission tomography (PET) imaging of somatostatin receptors (SST) has a high clinical impact on the management of neuroendocrine neoplasms (1, 2). Gallium-68 (^68^Ga)-labeled DOTA-Tyr^3^-octreotate (DOTA-TATE) (NETSPOT®) and DOTA-Tyr^3^-octreotide (DOTA-TOC) (SOMAKIT TOC®), where DOTA = 1,4,7,10-tetraazacyclododecane-1,4,7,10-tetraacetic acid, are two radiolabeled somatostatin analogs approved by the Food and Drug Administration (FDA) and the European Medicines Agency (EMA) for SST-PET imaging of neuroendocrine tumors (NETs). Kit-based formulation and an authorized ^68^Ge/^68^Ga-generator enable decentralized (on-site) preparation of these two radiotracers. This concept gained rapid adoption worldwide as it is easy to implement in clinical practice. However, there are certain limitations that are becoming increasingly apparent: (i) authorized generators are expensive and have a limited production capacity (the maximum number of patient doses per synthesis is 2–3, depending on the age of the generator), and (ii) access to gallium-68 is increasingly limited because another ^68^Ga-labeled tracer ([^68^Ga]Ga-PSMA-11, where PSMA = prostate specific membrane antigen) has also received FDA/EMA approval for PET imaging of prostate cancer and is in broad clinical use. Furthermore, the approved ^68^Ga-PET tracers have lutetium-177 (^177^Lu)-labeled therapeutic companions, i.e., [^177^Lu]Lu-DOTA-TATE (Lutathera®) and [^177^Lu]Lu-PSMA-617 (Pluvicto®). With SST- and PSMA-targeted radioligand therapy being important therapeutic options, the demand for ^68^Ga-PET scans is expected to expand rapidly. This demand is expected to further expand when new tracers are approved, for example, ^68^Ga-labeled fibroblast activation protein (FAP) inhibitors.

To address the clinical demand and upscaling of production, large-scale production of gallium-68 in cyclotrons has been explored but not implemented in clinical practice. Moreover, the short half-life of gallium-68 (t_1/2_ = 1.13 h) prevents the shipment of the radiotracer from central producers to smaller centers that are located beyond a 2 h journey away. Fluorine-18 (t_1/2_ = 1.83 h) addresses some of the hurdles of gallium-68. This comes, however, at the cost of facile chelator-based radiolabeling and the possibility of a therapeutic companion (theranostics); these are options that are possible with radiometals. Thus, somatostatin analogs labeled via aluminium-[^18^F]fluoride ([^18^F]AlF) were proposed, such as [^18^F]F-NOTA-octreotide (3), where NOTA = 1,4,7-triazacyclononane-1,4,7-triacetic acid, but these are limited to the chelator NOTA.

The cyclotron-produced *β*^+^ emitter copper-61 (^61^Cu, t_1/2_ = 3.33 h, E*_β_*^+^_max_ = 1.22 MeV, E*_β_*^+^_mean_ = 500 keV, *I_β_*^+^ = 61%) is a valuable alternative to gallium-68 (t_1/2_ = 1.13 h, E*_β_*^+^_max_ = 1.90 MeV, *I_β_*^+^ = 89%). Copper-61 can be produced in medical cyclotrons at a large scale and distributed to locations further away than gallium-68. Its lower positron energy (1.22 vs. 1.90 MeV) and mean range (1.3 vs. 2.4 mm in water) provide superior spatial resolution and improved imaging quality (4), suggesting enhanced detection of small lesions. Furthermore, its longer half-life allows imaging at multiple time points. This facilitates dosimetry estimates and potentially improves the identification of lesions due to the lower background over time. Last, but not least, copper-61 has a therapeutic twin, copper-67 [t_1/2_ = 2.58 days, E*_β_*^−^_max_ = 577 keV, E*_β_*^−^_mean_ = 141 keV, E*_γ_*_max_ = 185 keV (49%)]. Of note, even though copper-64 is more established for PET imaging, copper-61 offers valuable advantages that are addressed in the discussion part of the manuscript.

Despite the suitable physical characteristics of copper-61 for PET imaging, there are scarce reports on ^61^Cu-labeled tracers. The literature focuses mainly on production routes, separation methods, and optimization for high-purity copper-61 production. These have been the obstacles to overcome in the development of ^61^Cu-PET tracers. In 1999, McCarthy et al. utilized diacetyl-bis(*N^4^*-methylthiosemicarbazone (ATSM) and TETA-octreotide (where TETA = 1,4,8,11-tetraazacyclotetradecane-N,N’,N”,N”’-tetraacetic acid) to highlight the potential applications of copper-61 and copper-60 (5). Since then, only a very limited number of ^61^Cu-labeled tracers followed, primarily referring to ATSM (6, 7). Recently, ^61^Cu-labeled NAPamide for PET imaging of melanoma was reported (8, 9), while our group developed ^61^Cu-labeled PSMA and reported the first human ^61^Cu-PET (10). Today, large-scale production is feasible using either liquid targets via proton bombardment of natural zinc or enriched zinc-64 [^nat/64^Zn(p,*α*)^61^Cu] or using solid targets via deuteron bombardment of natural nickel or enriched nickel-61 [^nat/60^Ni(d,n)^61^Cu reaction] (11, 12). The optimization of separation and purification methods allows copper-61 production for direct radiolabeling and opens the way for the development of ^61^Cu-based PET tracers.

We explored the potential of copper-61 in combination with established somatostatin analogs given their proven clinical value in PET-SST imaging in neuroendocrine neoplasms. The GMP production of clinical doses of ^61^Cu-labeled DOTA-TATE and DOTA-TOC was recently reported by Fonseca et al., focusing on the radiochemical aspects (11). We report herein a comprehensive *in vitro* and *in vivo* evaluation of [^61^Cu]Cu-DOTA-TOC and its NODAGA (1,4,7-triazacyclononane,1-glutaric acid-4,7-acetic acid) derivative ([^61^Cu]Cu-NODAGA-TOC). The two ^61^Cu-labeled tracers were compared head-to-head with [^68^Ga]Ga-DOTA-TOC in terms of radiochemistry, affinity, and *in vitro* and *in vivo* performance.

## 2 Materials and methods

### 2.1 Reagents, instrumentation, and cell line

DOTA-TOC and NODAGA-TOC were purchased from piCHEM (Raaba-Grambach, Austria). All solvents and reagents were purchased and used as supplied by Sigma Aldrich (Switzerland) and VWR International (Switzerland) in high-performance liquid chromatography (HPLC) or analytical grades.

The human embryonic kidney (HEK) cell line expressing the T7-epitope-tagged human SST_2_ receptor (HEK-SST_2_) was provided by Prof. Stefan Schulz (Institute of Pharmacology and Toxicology, Jena University Hospital, Jena, Germany) and cultured at 37°C and 5% carbon dioxide (CO_2_) in Dulbecco's Modified Eagle Medium (DMEM) containing 10% fetal bovine serum (FBS), 100 U/ml penicillin, 100 mg/ml streptomycin, 200 µmol/ml L-Glutamin, and 500 mg/ml G418. All the reagents were purchased from BioConcept (Allschwill, Switzerland) and Biochrom GmbH, Merck Millipore (Darmstadt Germany).

Quantitative *γ*-counting was carried out on a Cobra 5003 *γ*-system well counter from Packard Instruments (Meriden, CT, USA).

### 2.2 ^61^Cu-labeled tracers and reference tracer

Copper-61 was produced by irradiating natural nickel electroplated on silver coins at 40 µA for 120 min in a GE HealthCare medical cyclotron at the University Hospital Zurich, Switzerland. Target dissolution and purification were based on Svedjehed et al. (12). A [^61^Cu]CuCl_2_ solution in 0.05 M HCl was produced at an activity concentration of approximately 1 GBq/ml for direct radiolabeling.

An aliquot of DOTA-TOC or NODAGA-TOC (3–6 nmol, 1 mg/ml in TraceSelect water) was diluted in 0.25–0.30 ml ammonium acetate (0.5 M, pH 8), followed by the addition of [^61^Cu]CuCl_2_ (30–150 MBq). The reaction mixture was incubated for 15 min at 95°C (DOTA-TOC) or at room temperature (NODAGA-TOC). The pH of the reaction mixture was between 5 and 6. Quality control was performed on a Shimadzu 2020 HPLC system connected to a Berthold radio detector (Flow Star 513). A sample of the labeling solution (1–5 μl) was withdrawn, diluted in 50 μl ethylenediaminetetraacetic acid (EDTA, 0.1 M), and analyzed on a Proteo Jupiter C12 (4.6 mm × 250 mm, 4 µm particle size), using a gradient of 15%–65% solvent *B* in 15 min [*A* = H_2_O (0.1% TFA), *B* = ACN (0.1% TFA)], at a flow rate of 1 ml/min. [^61^Cu]Cu-DOTA-TOC and [^61^Cu]Cu-NODAGA-TOC labeling solutions were stored at room temperature and analyzed up to 4 h after the end of synthesis by radio-HPLC.

The complexes of DOTA-TOC and NODAGA-TOC with natural copper (^nat^Cu) were prepared by incubating each conjugate (1–1.5 mg) with a 1.5-fold excess of ^nat^CuCl_2_ × 2 H_2_O in the same buffer used for radiolabeling, at 95°C for 30 min (DOTA-TOC) or 15 min (NODAGA-TOC). Uncomplexed natural copper ions were eliminated by SepPak C-18 purification. The ^nat^Cu-complexes were eluted with methanol followed by evaporation to dryness and lyophilization after dissolution in water. Analysis was performed via liquid chromatography-mass spectrometry (LC-MS) on an LCMS-2020 Shimadzu system equipped with a Waters X Bridge C18 column (4.6 mm × 150 mm, 5 µm particle size), using a gradient of 15%–65% solvent *B* for 15 min [*A* = H_2_O (0.1% TFA), *B* = ACN (0.1% TFA)] at a flow rate of 2 ml/min.

[^68^Ga]Ga-DOTA-TOC was synthesized in an automatic Modular-Lab Pharm Tracer module connected to a ^68^Ge/^68^Ga-generator IGG100 (Eckert & Ziegler), following the synthesis template and kit reagents of the manufacturer. ^nat^Ga-DOTA-TOC was synthesized using ^nat^Ga(NO_3_)_3_ × H_2_O and purified and analyzed as described above for the natural copper complexes.

### 2.3 Determination of lipophilicity

The lipophilicity of [^61^Cu]Cu-DOTA-TOC and [^61^Cu]Cu-NODAGA-TOC was assessed by the determination of the distribution coefficient (*D*), expressed as log *D* (pH = 7.4), between an aqueous and an organic phase following the “shake-flask” method. Briefly, the radiotracer (10 µl, 1 µM) was added to 1 ml of a 1:1 pre-saturated mixture of 1-octanol and phosphate-buffered saline (PBS pH 7.4). The solution was vortexed for 30 min and then centrifuged at 3,000 rpm to achieve phase separation. Aliquots of 0.1 ml from each phase were collected and measured in a *γ*-counter. The distribution coefficient was calculated as the average of the logarithmic values (*n* = 3) of the ratio between the radioactivity in the organic and PBS phases. [^68^Ga]Ga-DOTA-TOC was used as a reference.

### 2.4 Affinity studies

Competition binding and saturation binding experiments were conducted in HEK-SST_2_ cell membranes, and the data were analyzed using GraphPad Prism 9 software. The preparation of the cell membranes and the experimental details are as previously described (13).

For the competition binding experiments, membrane suspensions were incubated in 96 wells (10 µg/well) for 1 h at 37°C with the radioligand ^125^I-Tyr-SS-14 [Chelatec (Saint-Herblain, France), where SS-14 = somatostatin-14], at a concentration of 0.05 nM, and increasing concentrations of ^nat^Cu-DOTA-TOC or ^nat^Cu-NODAGA-TOC (ranging from 0.001 to 100 nM). Filtration on a Brandel 48-well Cell Harvester followed and the filtered membranes were measured in a *γ*-counter. ^nat^Ga-DOTA-TOC and SS-14 were used as references. The half-maximal inhibitory concentration (IC_50_) was determined using the “log(inhibitor) vs. response” equation.

The saturation binding experiments were performed to determine the dissociation constant (K_D_) and the maximum number of binding sites (B_max_). The cell membrane suspension was incubated with different concentrations (ranging from 0.075 to 10 nM) of ^61/nat^Cu-DOTA-TOC and ^61/nat^Cu-NODAGA-TOC for 1 h at 37°C, followed by membrane harvesting on a Brandel 48-well Cell Harvester, as described above.

In both experiments, non-specific binding was determined in the presence of a 1,000-fold excess of SS-14.

### 2.5 Cellular uptake and efflux

The cellular uptake and distribution of [^61^Cu]Cu-DOTA-TOC and [^61^Cu]Cu-NODAGA-TOC were evaluated on intact HEK-SST_2_ cells seeded in 6-well plates. The cells were each incubated with the radiotracer (2.5 nM/well) for 0.5, 1, 2, and 4 h at 37°C, either alone or in the presence of 1,000-fold excess of SS-14 to distinguish between specific and non-specific uptake. At the defined time points, the medium was removed, and the cells were washed twice with ice-cold PBS. The cells were then treated twice for 5 min with ice-cold glycine solution (0.05 M, pH 2.8) to detach the cell membrane-bound fraction (acid released). Afterward, the cells containing the internalized fraction were detached using NaOH 1 M at 37°C and collected for measurement. [^68^Ga]Ga-DOTA-TOC was evaluated as a reference.

The efflux was studied after 1 h incubation of the cells with the radiotracer at 37°C and the removal of the unbound fraction and the cell membrane-bound fraction, as described above (acid-wash). The cells were then incubated with fresh medium at 37°C either alone or with a competitor (1,000-fold excess of DOTA-TOC, 2.5 µM/well) for 10, 20, 30, 45, 60, 90, 120, 150, 180, and 240 min. At each time point, the efflux (or retention) was measured after the removal of the medium without or with the competitor and replacement with fresh pre-warmed (37°C) medium alone or with the competitor. At the end of the experiment, the cells were detached using 1 M NaOH at 37°C and collected to determine the remaining cell-associated radiotracer.

### 2.6 Animal studies

Animal experiments were conducted in accordance with Swiss animal welfare laws and regulations under license number 30515 granted by the Veterinary Office (Department of Health) of the Canton Basel-Stadt. Female athymic nude-*Foxn1^nu^/Foxn1^+^* mice (Envigo, The Netherlands), 4–6 weeks old, were inoculated subcutaneously with HEK-SST_2_ cells (10^7^ cells/100 μl), freshly suspended in sterile PBS, in the shoulder. The tumors were allowed to grow for 2–3 weeks, reaching a volume of 100–200 mm^3^.

### 2.7 PET/CT imaging studies

[^61^Cu]Cu-DOTA-TOC and [^61^Cu]Cu-NODAGA-TOC (100 µl/200 pmol/4–5 MBq) were intravenously injected in the tail vein of the HEK-SST_2_ xenografts. The mice were anesthetized with 1.5% isoflurane and dynamic PET scans were acquired from 0 to 1 h post-injection (p.i.). The mice were euthanized by CO_2_ at 4 h p.i., their bladders were mechanically emptied, and static PET scans were acquired for 30 min. PET scans of [^68^Ga]Ga-DOTA-TOC (100 µl/200 pmol/5 MBq) were acquired in a static mode 1 h p.i.

The PET images were acquired in list mode using a small-animal PET scanner (*β*-CUBE, Molecubes, Ghent, Belgium) with a spatial resolution of 0.85 mm and an axial field-of-view of 13 cm. All PET scans were decay corrected and reconstructed into a 192 × 192 × 384 matrix by an ordered subsets maximization expectation (OSEM) algorithm using 30 iterations, a voxel size of 400 µm × 400 µm × 400 µm, and15 min per frame. Computed tomography (CT) data was used to apply attenuation correction on the PET data. The CT was imaged supine, headfirst, using the nanoSPECT/CT™ scanner (Bioscan Inc.). Topograms and helical CT scans of the whole mouse were first acquired using the following parameters: x-ray tube current: 177 µA, x-ray tube voltage 45 kVp, 90 s, and 180 frames per rotation, pitch 1. The CT images were reconstructed using CTReco (version r1.146) with a standard filtered back projection algorithm (exact cone beam) and post-filtered (Ram-Lak, 100% frequency cut-off), resulting in a pixel size of 0.2 mm. Co-registered PET/CT images were visualized using maximum intensity projection (MIP) with VivoQuant software (version 4.0).

### 2.8 *In vivo* specificity

The specificity of the ^61^Cu-labeled tracers was assessed in HEK-SST_2_ xenografts by administering DOTA-TOC (100 µl/200 nmol) 2–5 min pre-injection, followed by the injection of the radiotracer (100 µl/200 pmol/4 MBq). The mice were euthanized 1 h p.i. and static PET/CT scans were performed, as described above.

### 2.9 Biodistribution

Quantitative biodistribution studies were performed for [^61^Cu]Cu-DOTA-TOC and [^61^Cu]Cu-NODAGA-TOC (100 µl/200 pmol/2–4 MBq) at 1 and 4 h p.i. The mice were euthanized at the time point of investigation by CO_2_ asphyxiation. Organs of interest and blood were collected, rinsed of excess blood, blotted dry, weighed, and counted in a *γ*-counter. The samples were counted against a suitably diluted aliquot of the injected solution as the standard. The results are expressed as a percentage of injected activity per gram of tissue (%IA/g) and represent the mean ± standard deviation (SD) of *n* = 4–9 mice/group. The biodistribution of [^68^Ga]Ga-DOTA-TOC was assessed at 1 h p.i. for comparison.

### 2.10 Dosimetry

Additional biodistribution data were generated in healthy BALB/c mice using [^64^Cu]Cu-NODAGA-TOC at 1, 4, 12, and 24 h p.i. and were combined with the data of [^61^Cu]Cu-NODAGA-TOC at 1 and 4 h p.i. [^64^Cu]CuCl_2_ was provided by the University Hospital Tübingen, Germany. The non-decay corrected biodistribution data for copper-61 (t_1/2_ = 3.33 h) were used to generate time-activity curves for [^61^Cu]Cu-NODAGA-TOC. OLINDA/EXM 1.0 was used to integrate the fitted time-activity curves and to estimate the organ and effective doses using the whole-body adult female model. For all the calculations, the assumption was made that the mouse biodistribution, determined as the %IA/organ, was the same as the human biodistribution. The dosimetry estimates were performed as previously described (14).

### 2.11 Statistics

Statistical analysis was performed by unpaired *t*-test with Welch's correction using GraphPad Prism software (GraphPad Inc., version 9). *P*-values < 0.05 were considered significant. All data were evaluated as mean ± standard deviation.

## 3 Results

### 3.1 ^61^Cu-labeled tracers and reference (radio)tracers

[^61^Cu]Cu-DOTA-TOC and [^61^Cu]Cu-NODAGA-TOC were synthesized with radiochemical purities of ≥96% and ≥98%, respectively, at an apparent molar activity of 25 MBq/nmol, without the need for a post-labeling purification step. [^61^Cu]Cu-DOTA-TOC required an elevated temperature (95°C), while [^61^Cu]Cu-NODAGA-TOC was synthesized at room temperature. Both ^61^Cu-labeled tracers remained stable in their buffered solution for up to 4 h at room temperature. The analytical data of the radiotracers are summarized in Table 1. [^68^Ga]Ga-DOTA-TOC was prepared at an apparent molar activity of 40 MBq/nmol and followed an established standard labeling procedure. Representative (radio)chromatograms of all radiotracers are provided in Supplementary Figure S1.

**Table 1:** Radiochemical purity, retention time (t_R_), and stability data of the ^61^Cu-labeled tracers.

Radiotracer	t_R_ (min)	Radiochemical purity (%)		
		T_0_	2 h	4 h
[^61^Cu]Cu-DOTA-TOC	9.8	98 ± 2	96 ± 1	95 ± 1
[^61^Cu]Cu-NODAGA-TOC	10.2	99 ± 1	98 ± 1	97 ± 1

The reference non-active complexes ^nat^Cu-DOTA-TOC and ^nat^Cu-NODAGA-TOC were prepared with a purity of >98% and characterized by reverse phase (RP)-HPLC and LC-MS. The analytical data are provided in Supplementary Figure S1 and Supplementary Table S1.

### 3.2 Determination of lipophilicity and *in vitro* characterization

The results of the log *D* determination and affinity measurements of [^61^Cu]Cu-DOTA-TOC and [^61^Cu]Cu-NODAGA-TOC, in comparison to [^68^Ga]Ga-DOTA-TOC, are summarized in Table 2.

**Table 2:** Lipophilicity and *in vitro* characteristics in HEK-SST_2_ cell membranes after 1 h incubation at 37°C.

Radiotracer	log *D*_(O/PBS pH 7.4)_	IC_50_ (nM)	K_D_ (nM)	B_max_ (nM)
^61/nat^Cu-DOTA-TOC	−2.81 ± 0.29	0.23 (0.21–0.26)	0.105 ± 0.011	0.093 ± 0.002
^61/nat^Cu-NODAGA-TOC	−2.60 ± 0.24	0.34 (0.30–0.38)	0.078 ± 0.007	0.064 ± 0.001
^68/nat^Ga-DOTA-TOC	−3.18 ± 0.11	0.18 (0.16–0.20)	n.d.	n.d.

Log *D*_O/PBS pH7.4_, log distribution coefficient between octanol and PBS phase 1:1 expressed as mean ± standard deviation; IC_50_, half-maximal inhibitory concentration, expressed as mean [95% confidence interval (CI)]; K_D_, dissociation constant; B_max_, maximum number of binding sites, as mean ± standard error, n.d. not determined.

The results were generated from of a minimum of two separate experiments, each in triplicate.

The labeling of DOTA-TOC with copper-61 vs. gallium-68 impacted lipophilicity (log *D* = −2.81 ± 0.29 vs. −3.18 ± 0.11, respectively). Among the three radiotracers, [^61^Cu]Cu-NODAGA-TOC was the most lipophilic (log *D* = −2.60 ± 0.24). Nevertheless, the log *D* values indicate that all three radiotracers are highly hydrophilic.

The two ^nat^Cu-complexes, ^nat^Cu-DOTA-TOC, and ^nat^Cu-NODAGA-TOC, showed a very high affinity for SST_2_ [IC_50_ = 0.23 nM (95% CI: 0.21–0.26) and 0.34 nM (95% CI: 0.30–0.38), respectively] and were in the same low sub-nanomolar level as the reference, ^nat^Ga-DOTA-TOC [IC_50_ = 0.18 nM (95% CI: 0.16–0.20)] and the SS-14 [IC_50_ = 0.11 nM (95% CI: 0.09–0.14)]. The saturation binding experiments showed no significant difference between ^61/nat^Cu-DOTA-TOC and ^61/nat^Cu-NODAGA-TOC by means of the maximum number of binding sites (B_max_) recognized by the radiotracers and their dissociation constant (K_D_). The results are summarized in Table 2. The dose–response curves from the competition binding experiments vs. ^125^I-Tyr-SS-14 and the saturation binding curves are shown in Supplementary Figures S2 and S3, respectively.

### 3.3 Cellular uptake and efflux

**Figure 1:**
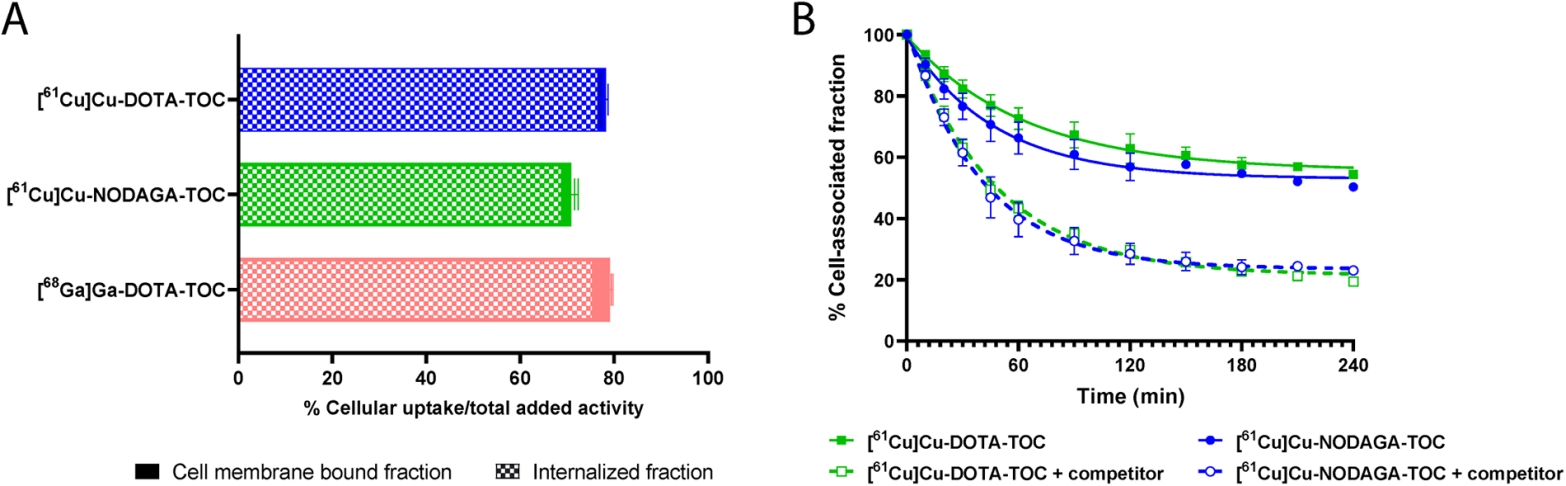
**(A)** Cellular uptake (surface bound + internalized) of the tested radiotracers in the HEK-SST_2_ cells after 4 h at 37°C. The results represent the mean ± standard deviation of the specific (=total − non-specific) uptake from a minimum of two separate experiments, each in triplicate. Cell membrane-bound and internalized fractions are indicated. **(B)** Efflux rate in the absence or in the presence of 1,000-fold excess of a competitor (DOTA-TOC) at 37°C. The data were analyzed according to the one-phase exponential decay equation (GraphPad Software Inc., Prism 9).

[^61^Cu]Cu-DOTA-TOC showed a higher cellular uptake than [^61^Cu]Cu-NODAGA-TOC (78.3 ± 1.3% and 70.9 ± 3.5%, respectively, after 4 h at 37°C, *p* = 0.0007) and was comparable to [^68^Ga]Ga-DOTA-TOC (79.0 ± 3.1%, after 4 h at 37°C, *p* = 0.59) (Figure 1A). The radiotracers were almost entirely internalized upon binding to SST_2_, while only a negligible amount remained on the cell membrane (<2% of the total added activity at 4 h). The cellular uptake and distribution over time are reported in Supplementary Figure S4. Blocking experiments with SS-14 proved the SST_2_-mediated uptake of both ^61^Cu-labeled tracers *in vitro*.

[^61^Cu]Cu-DOTA-TOC and [^61^Cu]Cu-NODAGA-TOC were externalized using a one-phase exponential decay model (Figure 1B). The efflux reached 80% after 4 h at 37°C when DOTA-TOC (competitor) was added to the medium. In the absence of a competitor, the efflux reached a plateau of approximately 40% after 2 h, without a further increase at 4 h, suggesting a rebinding of the externalized fraction of the radiotracer that remained in the proximity of the receptors.

### 3.4 PET/CT imaging studies

**Figure 2:**
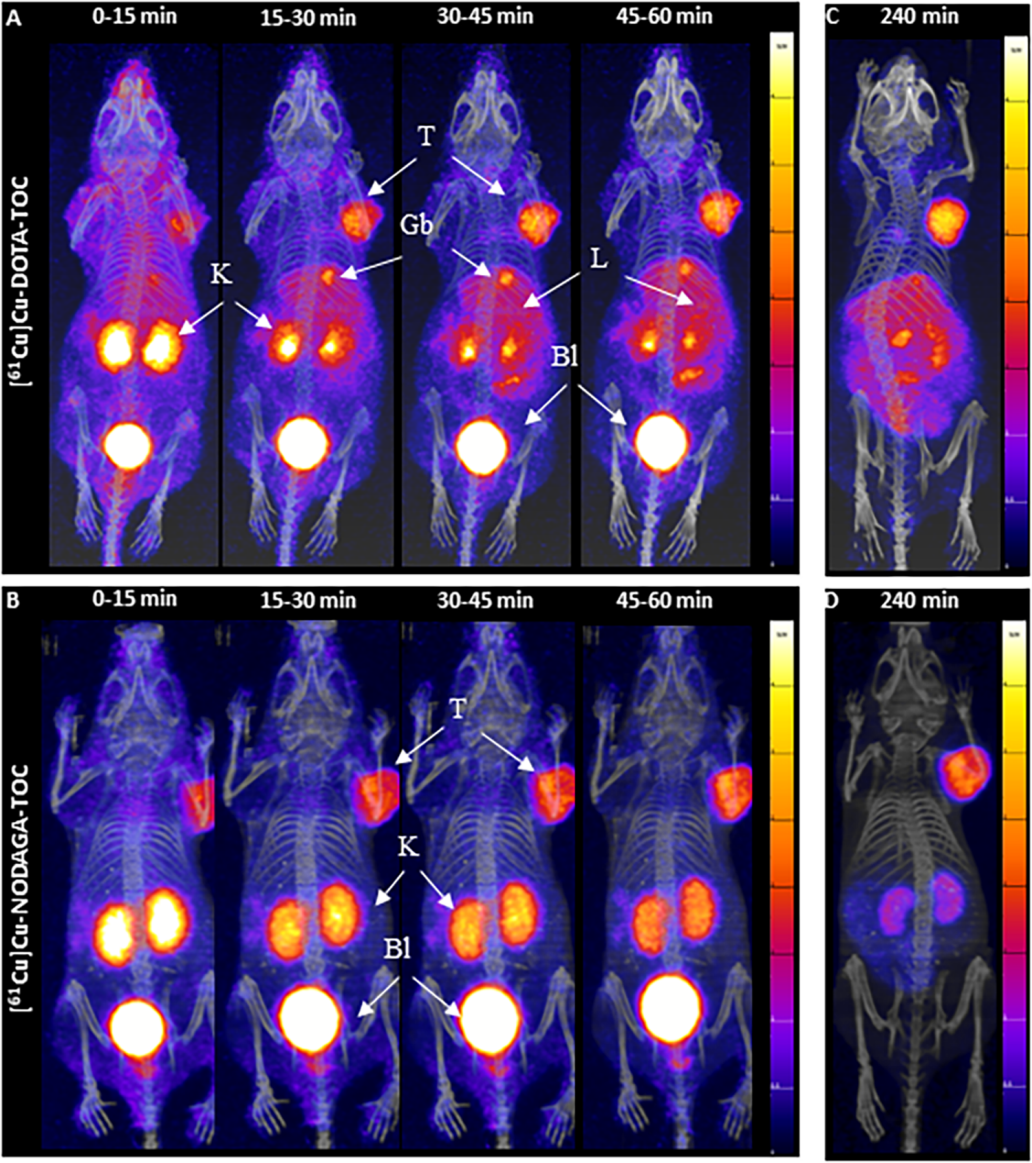
MIPs of the dynamic PET/CT scans of HEK-SST_2_ xenografts after an injection of 200 pmol/4-5mMBq [^61^Cu]Cu-DOTA-TOC **(A)** or [^61^Cu]Cu-NODAGA-TOC **(B)** from 0 to 1 h p.i., in 15 min frames. MIPs of static PET/CT scans at 4 h after an injection of 200 pmol/4-5 MBq [^61^Cu]Cu-DOTA-TOC **(C)** or [^61^Cu]Cu-NODAGA-TOC **(D)** into the HEK-SST_2_ xenografts. T, tumor; K, kidneys; L, liver; Gb, gallbladder; Bl, bladder; SUV, standard uptake value.

**Figure 3:**
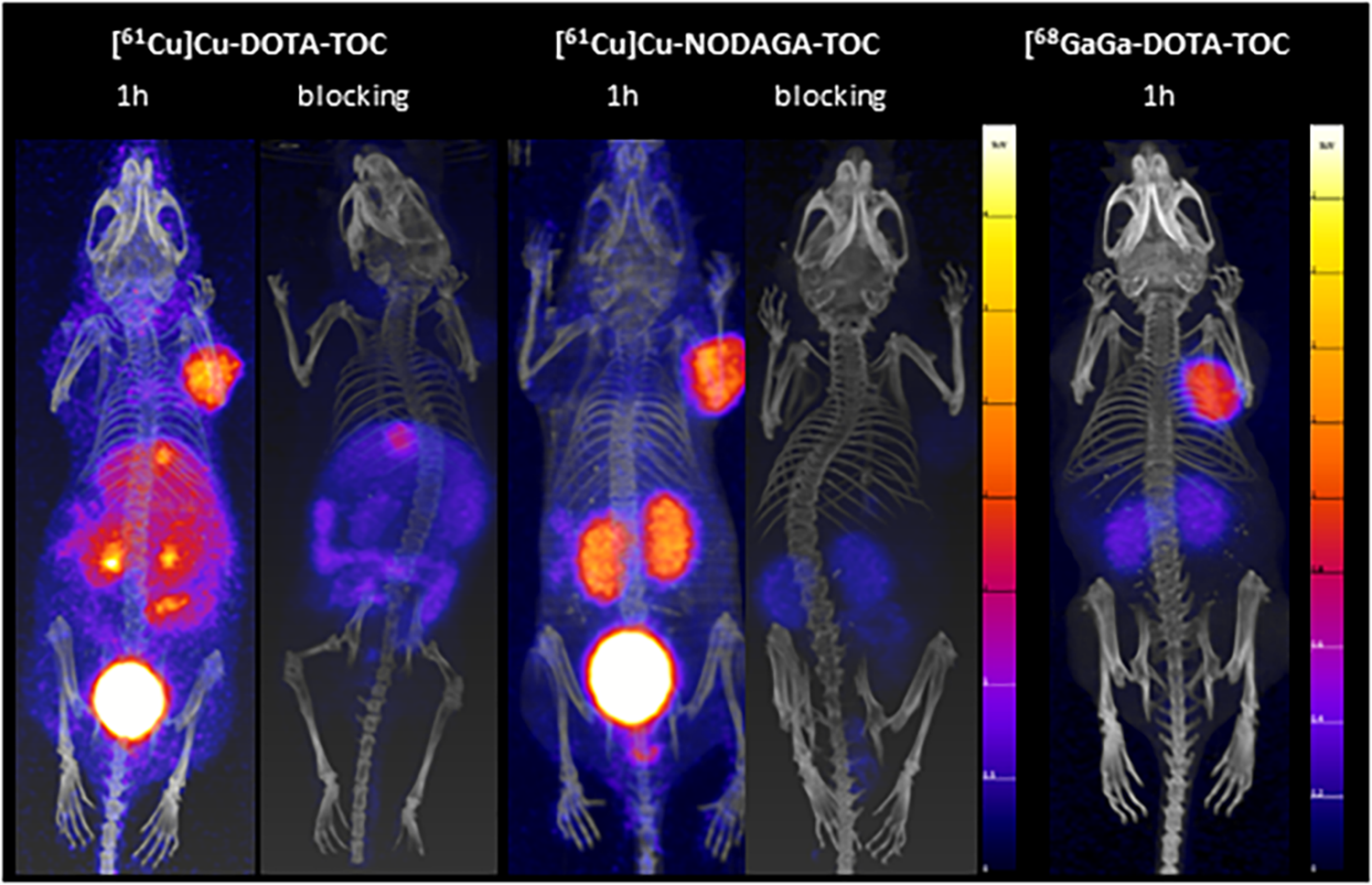
MIPs of the PET/CT scans of [^61^Cu]Cu-DOTA-TOC and [^61^Cu]Cu-NODAGA-TOC (100 µl/200 pmol/4-5 MBq) at 1 h p.i., with and without a pre-injection administration of a blocking agent (DOTA-TOC, 200 µmol). A PET/CT scan of [^68^Ga]Ga-DOTA-TOC (100 µl/200 pmol/5 MBq,) at 1 h p.i. is provided for visual comparison. The ^61^Cu-PET/CT scans were acquired in mice under anesthesia (full bladder), while the ^68^Ga-PET/CT scan was acquired in an euthanized mouse (bladder has been emptied mechanically). The PET images scale in 0-4 standardized uptake values (SUVs) for the ^61^Cu-PET images and 0-2 SUVs for the ^68^Ga-PET image.

Dynamic PET/CT images from between 0–1 h p.i. (Figure 2) revealed fast accumulation of [^61^Cu]Cu-DOTA-TOC and [^61^Cu]Cu-NODAGA-TOC in the tumor as early as 15 min p.i., and which further increased up to 1 h p.i. for both radiotracers. However, their total body distribution was distinctly different. [^61^Cu]Cu-DOTA-TOC accumulated rapidly in the liver, gallbladder, kidneys, and intestine (Figure 2A). In contrast, [^61^Cu]Cu-NODAGA-TOC accumulated primarily in the kidneys (Figure 2B). Over time, the uptake of [^61^Cu]Cu-DOTA-TOC in the abdomen remained persistent, while no improvement of the tumor-to-background contrast was observed. In contrast, the tumor-to-non-tumor ratios were improved for [^61^Cu]Cu-NODAGA-TOC, especially the tumor-to-kidney ratio (see Figures 2C,D, respectively). PET/CT imaging of [^61^Cu]Cu-NODAGA-TOC compared well with the reference, [^68^Ga]Ga-DOTA-TOC, at 1 h p.i., while [^61^Cu]Cu-DOTA-TOC was clearly inferior (Figure 3).

High excess DOTA-TOC pre-injection resulted in a significant reduction of the accumulation of both ^61^Cu-labeled tracers in the tumors and SST_2_-positive organs, confirming their receptor-mediated uptake in these tissues (Figure 3). Nevertheless, the abdominal uptake of [^61^Cu]Cu-DOTA-TOC, especially in the liver and intestine, was not suppressed, suggesting that this uptake was non-specific. The relatively high liver and intestine uptake of [^61^Cu]Cu-DOTA-TOC can be attributed, at least partially, to the release of copper-61 from the [^61^Cu]Cu-DOTA complex and the bio-reduction of Cu(II) to Cu(I) that can be incorporated into Cu-binding proteins (15).

### 3.5 Biodistribution studies

The biodistribution data are presented in Table 3. [^61^Cu]Cu-DOTA-TOC and [^61^Cu]Cu-NODAGA-TOC showed similar tumor uptake at 1 h p.i. (7.44 ± 2.33%IA/g and 8.88 ± 3.19%IA/g, respectively, *p* = 0.46) and at 4 h p.i. (6.85 ± 2.48%IA/g and 7.39 ± 1.36%IA/g, *p* = 0.67, respectively). Tumor uptake remained the same between 1 and 4 h p.i. for both the radiotracers (*p* = 0.72 for [^61^Cu]Cu-DOTA-TOC and *p* = 0.37 for [^61^Cu]Cu-NODAGA-TOC).

**Table 3:** Biodistribution of [^61^Cu]Cu-DOTA-TOC, [^61^Cu]Cu-NODAGA-TOC, and [^68^Ga]Ga-DOTA-TOC in the HEK-SST_2_ xenografts.

Organ	[^61^Cu]Cu-DOTA-TOC		[^61^Cu]Cu-NODAGA-TOC		[^68^Ga]Ga-DOTA-TOC
	1 h	4 h	1 h	4 h	1 h
Blood	0.38 ± 0.09	0.21 ± 0.04	0.22 ± 0.04	0.04 ± 0.02	0.63 ± 0.09
Heart	0.63 ± 0.07	0.49 ± 0.06	0.16 ± 0.04	0.07 ± 0.03	0.27 ± 0.03
Lung	1.96 ± 0.16	1.25 ± 0.08	1.09 ± 0.20	0.51 ± 0.24	1.68 ± 0.32
Liver	3.59 ± 0.49	2.52 ± 0.56	0.29 ± 0.04	0.27 ± 0.09	0.58 ± 0.07
Pancreas	4.29 ± 0.50	0.68 ± 0.07	2.59 ± 0.49	0.60 ± 0.21	4.77 ± 1.16
Spleen	0.55 ± 0.08	0.37 ± 0.09	0.22 ± 0.05	0.10 ± 0.04	0.42 ± 0.05
Stomach	4.34 ± 0.50	2.09 ± 0.50	2.34 ± 0.43	1.22 ± 0.25	3.73 ± 0.36
Intestine	2.94 ± 0.15	2.06 ± 0.92	0.92 ± 0.12	0.65 ± 0.23	1.39 ± 0.28
Adrenal	2.05 ± 0.58	1.14 ± 0.50	0.99 ± 0.17	0.67 ± 0.26	2.57 ± 0.78
Kidneys	5.32 ± 0.58	2.33 ± 0.39	12.5 ± 2.25	4.36 ± 0.92	8.37 ± 0.84
Muscle	0.19 ± 0.05	0.16 ± 0.08	0.16 ± 0.06	0.07 ± 0.04	0.23 ± 0.09
Bone	0.60 ± 0.16	0.50 ± 0.19	0.46 ± 0.17	0.31 ± 0.11	0.49 ± 0.07
Pituitary	3.00 ± 1.32	2.43 ± 1.27	3.80 ± 1.35	2.97 ± 0.95	3.29 ± 0.63
SST_2_-tumor	7.44 ± 2.33	6.85 ± 2.48	8.88 ± 3.19	7.39 ± 1.36	6.64 ± 1.11
Ratios					
Tumor/blood	20	33	40	185	11
Tumor/liver	2.1	2.7	31	27	12
Tumor/kidney	1.4	2.9	0.7	1.7	0.8
Tumor/muscles	39	43	56	106	29

Results are expressed as the mean of the percentage of injected activity per gram of tissue (%IA/g) ± standard deviation (SD) of *n* = 4–9 mice/group.

Distinct differences were observed between the two radiotracers in their total body distribution, especially in the liver, abdomen, and kidneys. At the early time point of investigation (1 h p.i.), [^61^Cu]Cu-DOTA-TOC showed a significantly lower uptake in the kidneys compared with [^61^Cu]Cu-NODAGA-TOC (5.32 ± 0.58%IA/g vs. 12.5 ± 2.25%IA/g, *p* = 0.0013), but a significantly higher uptake in the liver (3.59 ± 0.49%IA/g vs. 0.29 ± 0.04%IA/g, *p* = 0.0009) and intestine (2.94 ± 0.15%IA/g vs. 0.92 ± 0.12%IA/g, *p* < 0.0001). At the late time point of investigation (4 h p.i.), these differences remained comparatively the same. Despite the washout from all organs but the tumors, [^61^Cu]Cu-DOTA-TOC retained a clearly higher background activity, especially in the abdomen, and ∼5-fold higher blood values compared with [^61^Cu]Cu-NODAGA-TOC. In contrast, [^61^Cu]Cu-NODAGA-TOC had a fast blood and body clearance, with persistent uptake mainly in the kidneys and tumor.

With the exception of the tumor-to-kidney ratio, all the other tumor-to-non-tumor ratios, including the tumor-to-blood ratio, were in favor of [^61^Cu]Cu-NODAGA-TOC. All ratios, including the tumor-to-kidneys ratio, were significantly improved from 1 to 4 h p.i., due to the background clearance and the persistent tumor uptake.

[^61^Cu]Cu-DOTA-TOC showed very similar accumulation in the SST_2_-expressing tissues (e.g., tumor, stomach, or pancreas) as the reference radiotracer [^68^Ga]Ga-DOTA-TOC (Table 3). However, as described above, it had significantly higher liver (3.59 ± 0.49%IA/g vs. 0.58 ± 0.07%IA/g, *p* = 0.001) and intestinal (2.94 ± 0.15%IA/g vs. 1.39 ± 0.28%IA/g, *p* = 0.0003) uptake. [^61^Cu]Cu-NODAGA-TOC had a similar uptake in the tumor (8.88 ± 3.19%IA/g vs. 6.64 ± 1.11%IA/g, *p* = 0.207) and a higher uptake in the kidneys (12.5 ± 2.25%IA/g vs. 8.37 ± 0.84%IA/g, *p* = 0.011) compared with [^68^Ga]Ga-DOTA-TOC at 1 h p.i. [^61^Cu]Cu-NODAGA-TOC showed improved tumor-to-non-tumor ratios for all the considered organs, except the kidneys. This advantage can be attributed to copper-61 and/or to the conjugate NODAGA-TOC, thus it is potentially possible to also gain this advantage with [^68^Ga]Ga-NODAGA-TOC. However, [^61^Cu]Cu-NODAGA-TOC demonstrated the best ratios at 4 h p.i., (a time point compatible with the physical half-life of copper-61, but not that of gallium-68) among the three radiotracers and the two time points of investigation.

### 3.6 Dosimetry

The pharmacokinetic data of [^61/64^Cu]Cu-NODAGA-TOC from 1 up to 24 h p.i. are provided in Supplementary Table S2 and were used for the dosimetry estimates. Table 4 shows the estimated radiation dose of [^61^Cu]Cu-NODAGA-TOC for human females with an effective dose of 0.00241 mSv/MBq.

**Table 4:** Total absorbed doses of [^61^Cu]Cu-NODAGA-TOC in different organs calculated by OLINDA/EXM version 1.0 with a standard adult female phantom.

Target organ	Total absorbed dose (mGy/MBq)
Adrenals	6.77 × 10^−3^
Brain	1.53 × 10^−5^
Breasts	2.14 × 10^−4^
Gallbladder wall	2.14 × 10^−3^
LLI wall	8.28 × 10^−4^
Small intestine	1.12 × 10^−2^
Stomach wall	5.34 × 10^−3^
ULI wall	2.14 × 10^−3^
Heart wall	1.02 × 10^−3^
Kidneys	9.65 × 10^−2^
Liver	4.06 × 10^−3^
Lungs	1.67 × 10^−3^
Muscle	5.63 × 10^−4^
Ovaries	1.19 × 10^−3^
Pancreas	2.23 × 10^−2^
Red marrow	1.07 × 10^−3^
Osteogenic cells	6.57 × 10^−4^
Skin	2.42 × 10^−4^
Spleen	3.49 × 10^−3^
Thymus	1.98 × 10^−4^
Thyroid	5.27 × 10^−5^
Urinary bladder wall	3.48 × 10^−4^
Uterus	1.03 × 10^−3^
Total body	1.27 × 10^−3^
Effective dose (mSv/MBq)	2.41 × 10^−3^

LLI, Lower Large Intestine; ULI, Upper Large Intestine.

## 4 Discussion

In the era of precision medicine, patient-tailored treatments rely on advanced imaging tools. A neuroendocrine tumor is an exemplary case where precision medicine is realistic via PET/CT imaging with radiolabeled somatostatin analogs. This study aimed to provide insights into the advantages of ^61^Cu-labeled somatostatin analogs in comparison with the established ^68^Ga-labeled analogs for PET imaging of SST-expressing tumors. Furthermore, it aimed to compare copper-61 and copper-64 in view of the recently FDA-approved [^64^Cu]Cu-DOTA-TATE (Detectnet®) and other ^64^Cu-labeled somatostatin analogs.

In this study, copper-61 was produced in a medical cyclotron after deuteron irradiation of a natural nickel solid target. Target dissolution and purification were adapted and adjusted from Svedjehed et al. (12) and the details will be published elsewhere (manuscript in preparation). Copper-61 was produced with a radionuclidic purity exceeding 99.99% at 12 h post-synthesis and in a very high purity for direct radiolabeling. [^61^Cu]Cu-DOTA-TOC and [^61^Cu]Cu-NODAGA-TOC were synthesized with high radiochemical purities (≥96% and ≥98%, respectively), at an apparent molar activity of 25 MBq/nmol without the need for a post-labeling purification step. ^61^Cu-labeling of the SST analogs DOTA-TOC, DOTA-TATE, and DOTA-NOC with a labeling yield >97% has been reported in the literature when using copper-61 produced from an enriched zinc-64 liquid target (11). The use of an inexpensive natural nickel solid target to produce high-purity copper-61 reported in this study is economically more sustainable and, thus, more attractive.

In comparison with the reference, [^68^Ga]Ga-DOTA-TOC, the two ^61^Cu-tracers were more lipophilic (*p* < 0.0001 for [^61^Cu]Cu-DOTA-TOC vs. [^68^Ga]Ga-DOTA-TOC and *p* < 0.0001 for [^61^Cu]Cu-NODAGA-TOC vs. [^68^Ga]Ga-DOTA-TOC). The *in vitro* assessment of the SST_2_-expressing intact cells and membranes did not indicate significant differences between the two ^61^Cu-tracers and in comparison with [^68^Ga]Ga-DOTA-TOC. However, significant differences were observed among the three radiotracers *in vivo*. More specifically, while the tumor uptake of TOC was not impacted by the radionuclide (copper-61 or gallium-68) or the chelator (DOTA or NODAGA), the distribution in the other organs was significantly impacted. In comparison with [^68^Ga]Ga-DOTA-TOC, [^61^Cu]Cu-DOTA-TOC showed major differences, while [^61^Cu]Cu-NODAGA-TOC showed a similar biodistribution pattern at 1 h p.i. In contrast with the other two, [^61^Cu]Cu-DOTA-TOC showed unfavorable biodistribution, characterized by high and persistent accumulation in the liver and the abdomen, which are proven to be non-SST-mediated (see specificity studies). The results indicate that ^61^Cu-based PET tracers using DOTA as a chelator have an unfavorable biodistribution.

Though, when [^64^Cu]Cu-DOTA-TATE was compared with [^68^Ga]Ga-DOTA-TOC in the same NET patients, it showed the same sensitivity on a patient-by-patient basis and a higher lesion detection rate (16). However, this was not attributed to the radiotracer itself but mainly to the shorter *β*^+^ range of copper-64 compared with gallium-68. Currently, there are no conclusive data on whether [^64^Cu]Cu-DOTA-TATE or [^68^Ga]Ga-DOTA-TOC/-TATE is superior (17). Moreover, it remains unclear whether the liver uptake of [^64^Cu]Cu-DOTA-TATE is a limitation in detecting lesions, especially in the liver, the primary organ of metastasis of NETs. The liver uptake of ^64^Cu-tracers or other ^x^Cu-based radiopharmaceuticals, especially those based on DOTA and DOTA-derivatives, is mainly attributed to the biologically triggered reduction of Cu(II) to Cu(I) and the inability of DOTA to stabilize Cu(I), leading to accumulation in the liver and other off-target tissues (10, 18–20). In our recent study, in which the *in vivo* metabolic stability of [^61^Cu]Cu-NODAGA-PSMA and [^61^Cu]Cu-DOTAGA-PSMA was investigated, we demonstrated that a substantial amount of copper-61 was released from the DOTAGA-complex and accumulated in the liver and the abdomen, similar to [^61^Cu]CuCl_2_ (10). These common biodistribution features of [^61^Cu]Cu-DOTAGA-PSMA and [^61^Cu]CuCl_2_ were also observed in the biodistribution of [^61^Cu]Cu-DOTA-TOC, suggesting the release of copper-61. Among the alternative chelators developed for copper, the cage amine ligand MeCOSAR {5-[8-methyl-3,6,10,13,16,19-hexaaza-bicyclo(6.6.6)icosan-1-ylamino]-5-oxopentanoic acid}, called sarcophagine (Sar), was used successfully in combination with TATE. In comparison with [^68^Ga]Ga-DOTA-TATE, [^64^Cu]Cu-SAR-TATE had very similar outcomes in the PET images of NET patients at 1 h p.i., and clear advantages at 4 and 24 h p.i. compared with 1 h p.i. due to the increased lesion-to-liver ratios (21).

Our findings with [^61^Cu]Cu-NODAGA-TOC are in line with the clinical observations of [^64^Cu]Cu-SAR-TATE, supporting the advantages of imaging at a later time point. [^61^Cu]Cu-NODAGA-TOC had a fast blood and background clearance, with persistent uptake mainly in the kidneys and tumor. With the exception of the tumor-to-kidney ratio, all the other tumor-to-non-tumor ratios and the tumor-to-blood ratio were in favor of [^61^Cu]Cu-NODAGA-TOC at 1 h p.i. and they were all, including the tumor-to-kidney ratio, significantly improved from 1 h to 4 h p.i. due to the background clearance and the persistent tumor uptake. Furthermore, the tumor uptake of the radiolabeled somatostatin analogs continued to increase up to approximately 12 h p.i (22). Even though an uptake time of 1 h p.i. is recommended for ^68^Ga-PET scans (23), there are indications that late-time imaging improves the detection rate (21, 24, 25). This might be valuable for unclear or difficult cases, especially regarding liver lesions. The half-life of copper-61 is compatible with the pharmacokinetics of somatostatin analogs and with early- and late-time imaging, i.e., 1 and 3–4 h after injection. Furthermore, NODAGA is a chelator that forms a stable complex with Cu(II), in contrast with DOTA, thus liver uptake is not of concern. Furthermore, NODAGA has the advantage of fast kinetics and radiolabeling at room temperature within a few minutes. These characteristics are very attractive for kit formulation and routine daily clinical use.

In principle, copper-64 also allows for late-time imaging and alleviates central production and shipment issues when compared with the logistical challenges of gallium-68. However, there are two main reasons to consider copper-61 over copper-64. The first is the positron-branching fraction as it is only 18% for copper-64 vs. 61% for copper-61. This implies that copper-64 requires a much higher injected dose or longer scanning time than copper-61 to obtain the same photon count statistics, while the image reconstruction parameters might be challenging. The low positron-branching fraction in combination with the longer half-life of copper-64 (t_1/2_ = 12.7 h) and its *β*^−^ component (39%) account for the higher level of radiation exposure with copper-64. The reported effective dose of [^64^Cu]Cu-DOTA-TATE in humans ranges from 0.0315 to 0.0454 mSv/MBq (21, 26), while for [^68^Ga]Ga-DOTA-TOC or [^68^Ga]Ga-DOTA-TATE it is 0.021–0.0257 mSv/MBq (27, 28). This difference increases due to the higher injected activity demand in the case of copper-64. In our study, we used biodistribution data from mice and assumed that humans have the same pharmacokinetics. [^61^Cu]Cu-NODAGA-TOC had an effective dose of 0.00241 mSv/MBq and a total-body absorbed dose of 1.27 × 10^−3^ mGy/MBq, while the organ with the highest dose was the kidneys (9.65 × 10^−2^ mGy/MBq). In comparison with the dosimetry data for [^68^Ga]Ga-DOTA-TATE extrapolated from mice for humans (e.g., a total-body absorbed dose of 1.27 × 10^−3^ mGy/MBq and kidney dose of 9.01 × 10^−2^ mGy/MBq) (29), the data confirmed that copper-61 is within the safe radiation limits for PET imaging.

The second argument for copper-61 vs. copper-64 is related to the production. Copper-61 can be produced through the deuteron bombardment of natural nickel. For increased production volumes, this process can also utilize isotopically enriched nickel-60 or nickel-61. The yield of copper-61 can vary from 3 to 100 GBq, depending on the enrichment of the starting nickel material and beam parameters. This variability enables the adjustment of production to meet specific patient demands. Generating copper-61 necessitates 1–3 h of cyclotron beam time and an additional 30 min for purification. Conversely, copper-64 necessitates 4–12 h of beam time, with lower yields ranging from 3 to 10 GBq. The extensive beam time makes copper-64 production less practical for busy hospitals or manufacturing organizations involved in the production of multiple radiopharmaceuticals. Furthermore, the use of highly enriched (>98%) nickel-64 is required for copper-64 production to achieve the necessary radionuclidic purity and specific activity. Given that the cost of the target material and beam time are significant contributors to the overall production cost, the scalability of copper-61 production from an economic standpoint is more attractive.

Overall, copper-61 is a PET radionuclide with balanced characteristics and logistics that is between the short-lived, high-energy gallium-68 and the long-lived, low-energy copper-64 (4). In the era of radio-theranostics, research on new or unexplored radionuclides—especially in view of new discoveries, high clinical demand, and shortage of different radionuclides—is more pressing than ever. Radiometals are major players in this era, especially those with theranostic twins. Copper-61/copper-67 are among these major players.

## 5 Conclusion

Somatostatin receptor ^61^Cu-PET imaging not only matches the performance of ^68^Ga-PET at 1 h p.i. but has advantages in late-time imaging at 4 h p.i. This improves image contrast due to the increased tumor-to-non-tumor ratios, allows flexibility, and provides options for dosimetry estimates. [^61^Cu]Cu-NODAGA-TOC is superior to [^61^Cu]Cu-DOTA-TOC *in vivo*. The use of the chelator NODAGA allows quantitative labeling with copper-61 at room temperature and enables the straightforward use of kit formulation for simple manufacturing in medical centers. Copper-61 has advantages over copper-64 in terms of dosimetry and production logistics. Copper-61 and its therapeutic twin copper-67 comprise an ideal theranostic pair for Cu-based radiopharmaceuticals.

## Data Availability

The raw data supporting the conclusions of this article will be made available by the corresponding author on reasonable request.
