# Supplementary material for: Copper-61 is an advantageous alternative to gallium-68 for PET imaging of somatostatin receptor-expressing tumors: a head-to-head comparative preclinical study
Source: Front Nucl Med. 2024 Oct 11;4:1481343. doi: 10.3389/fnume.2024.1481343 (PMC11503464; doi:10.3389/fnume.2024.1481343)
Supplement: Supplementary file 1 [file Datasheet1.pdf]

Supplemental Information

^{61}Cu is an advantageous alternative to ^{68}Ga for PET imaging of somatostatin receptor expressing tumors: a head-to-head comparative preclinical study

1 Supplementary Figures and Tables

1.1 Supplementary Figures

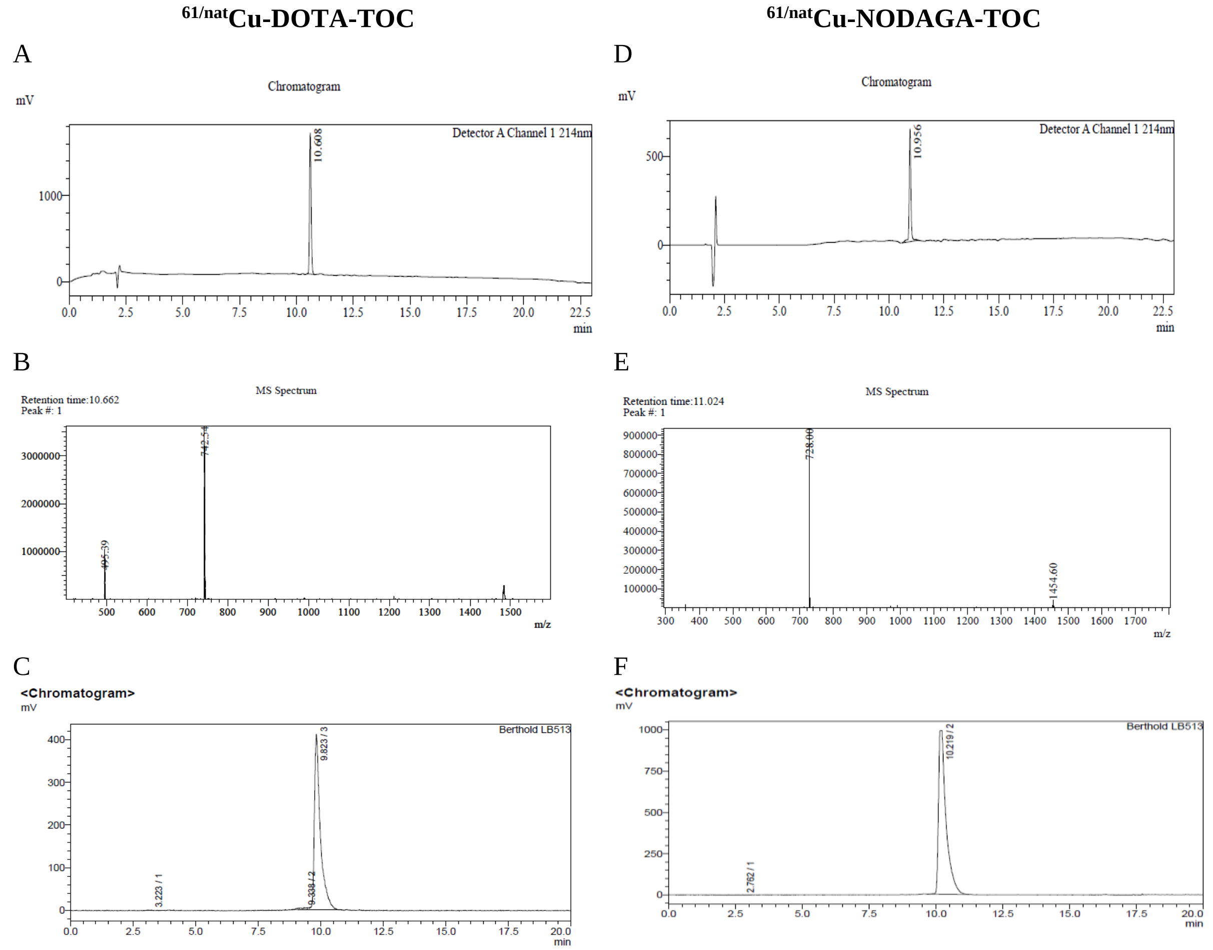

SUPPL. FIGURE S1. UV-chromatograms and mass spectrum of ^{nat}Cu-DOTA-TOC (A and B, respectively) and ^{nat}Cu-NODAGA-TOC (D and E, respectively), analyzed on a LCMS-2020 Shimadzu system. Radio-chromatograms of [^{61}Cu]Cu-DOTA-TOC (C) and [^{61}Cu]Cu-NODAGA-TOC (F) analyzed on a Shimadzu 2020 system, as described in the Materials and methods.

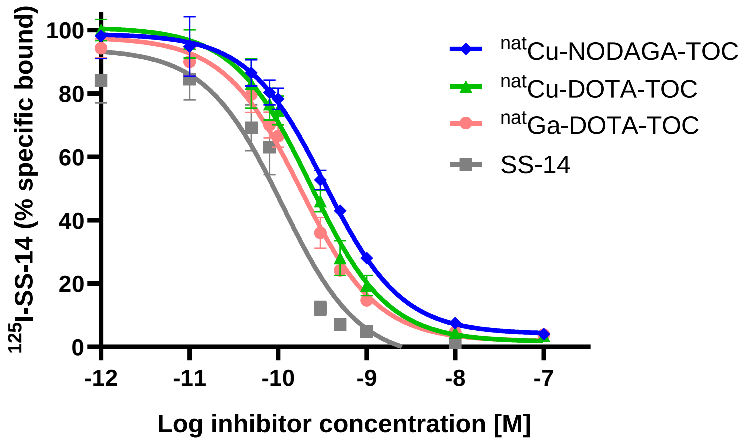

SUPPL. FIGURE S2. Competition binding activity curves of ^{nat}Cu-DOTA-TOC, ^{nat}Cu-NODAGA-TOC, compared to ^{nat}Ga-DOTA-TOC and SS-14 on HEK-SST$_2$ membranes after 1 hour incubation at 37°C using ^{125}I-Tyr-SS-14 as reference radioligand. The results are expressed as means ± standard deviation (SD) from a minimum of two separate experiments, each in triplicates and were calculated using the 'log(inhibitor) *vs* response' equation (GraphPad Software Inc., Prism 9).

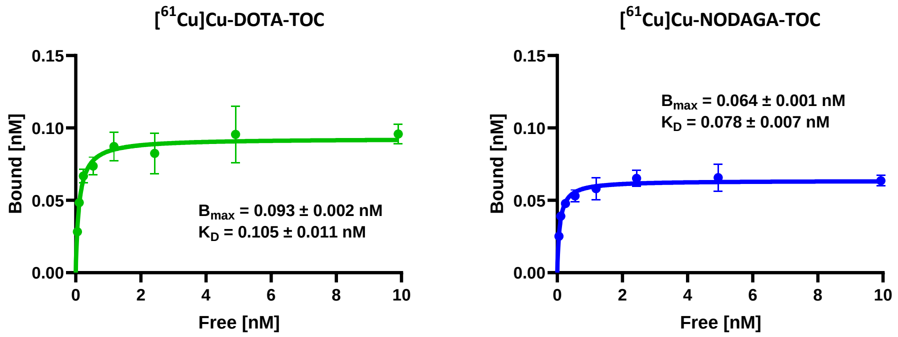

SUPPL. FIGURE S3. Saturation binding curves plotted as specific bound vs free fractions of $^{61/nat}$Cu-DOTA-TOC and $^{61/nat}$Cu-NODAGA-TOC after incubation of increasing concentrations with HEK-SST$_2$ membrane at 1 hour at 37°C. The values as expressed as mean ± standard deviation (SD) from a minimum of two separate experiments, each in triplicate and analyzed using GraphPad Software Inc., Prism 9).

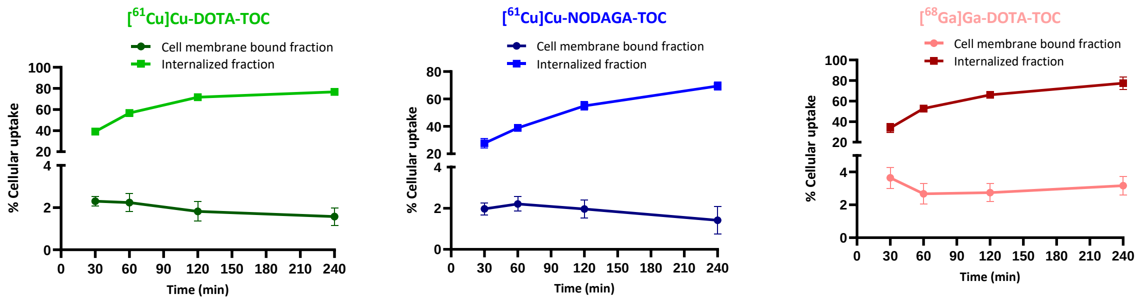

SUPPL. FIGURE S4. Cellular uptake and distribution between cell membrane bound and internalized fractions of the ^{61}Cu-labeled tracers, in comparison to [^{68}Ga]Ga-DOTA-TOC. The values are expressed as % of the applied activity and refer to the specific uptake calculated after subtracting the non-specific uptake (measured in the presence of 1,000-fold excess of SS-14) from the total uptake (specific = total – nonspecific). The results are from minimum two separate experiments per radiotracer, each in triplicates.

1.2 Supplementary Tables

SUPPL. TABLE S1. Analytical data of ^{nat}Cu- and ^{nat}Ga-complexed ligands.

Metallated ligand	m/z (calculated)	m/z (observed)	t_R (min)
^{nat}Cu-DOTA-TOC	742.6	742.5	10.61
^{nat}Cu-NODAGA-TOC	728.1	728.0	10.96
^{nat}Ga-DOTA-TOC	745.2	745.5	10.46

m/z: mass-to-charge ratio of the ion $[M+2H]^{2+}$, t_R: retention time

SUPPL. TABLE S2. Biodistribution of [$^{61/64}$Cu]Cu-NODAGA-TOC at 1h and 4h p.i. in Balb/c and HEK-SST$_2$ xenografts (combined, n=10-12/group) and [^{64}Cu]Cu-NODAGA-TOC at 12h and 24h p.i. in Balb/c mice (n=5/group).

Organ	1 h	4 h	12 h	24 h
Blood	0.24±0.08	0.04±0.02	0.04±0.01	0.03±0.00
Heart	0.17±0.03	0.07±0.02	0.09±0.02	0.07±0.02
Lung	1.07±0.17	0.50±0.24	0.51±0.12	0.27±0.09
Liver	0.35±0.08	0.28±0.08	0.37±0.09	0.26±0.02
Pancreas	2.71±0.40	0.60±0.19	0.14±0.03	0.07±0.01
Spleen	0.23±0.04	0.10±0.03	0.10±0.02	0.07±0.02
Stomach	2.62±0.48	1.35±0.26	0.75±0.19	0.26±0.04
Intestine	0.96±0.12	0.69±0.19	0.57±0.07	0.31±0.05
Adrenal	1.06±0.24	0.63±0.22	0.32±0.20	0.18±0.10
Kidneys	17.2±4.46	7.64±3.85	2.46±0.85	0.66±0.15
Muscles	0.16±0.05	0.07±0.03	0.02±0.01	0.01±0.00
Femur	0.45±0.14	0.28±0.11	0.11±0.04	0.06±0.01
Pituitary	3.39±1.11	2.71±1.84	0.48±0.25	0.43±0.29

Results are expressed as mean of the percentage injected activity per gram of tissue (%IA/g) ± standard deviation (SD).
